# Changes in human walking dynamics induced by uneven terrain are reduced with ongoing exposure, but a higher variability persists

**DOI:** 10.1038/s41598-019-54050-z

**Published:** 2019-11-27

**Authors:** Jenny A. Kent, Joel H. Sommerfeld, Nicholas Stergiou

**Affiliations:** 1 Division of Biomechanics and Research Development, University of Nebraska at Omaha, 6160 University Drive, Omaha, NE 68182-0860 USA; 2 College of Public Health, 984355 University of Nebraska Medical Center, Omaha, NE 68198-4355 USA; 3 Present Address: Feinberg School of Medicine, Northwestern University, Suite 1100, 680 N Lake Shore Drive, Chicago, IL 60611 USA

**Keywords:** Behavioural methods, Motor control, Dynamical systems

## Abstract

During walking, uneven terrain alters the action of the ground reaction force from stride to stride. The extent to which such environmental inconsistencies are withstood may be revealed by the regulation of whole-body angular momentum (*L*) during walking. *L* quantifies the balance of momenta of the body segments (thigh, trunk, etc.) about their combined center of mass, and remains close to zero during level walking. A failure to constrain *L* has been linked to falls. The aim of this study was to explore the ability of young adults to orchestrate their movement on uneven terrain, illustrated by the range of *L* (*L*_R_) and its variability (v*L*_R_). In eleven male adults, we observed significant increases in sagittal plane *L*_R_, and v*L*_R_ in all three planes of motion during walking on an uneven in comparison to a flat surface. No reductions in these measures were observed within a 12-minute familiarisation period, suggesting that unimpaired adults either are unable to, or do not need to eliminate the effects of uneven terrain. Transverse plane *L*_R_, in contrast, was lower on immediate exposure, and then increased, pointing to the development of a less restrictive movement pattern, and would support the latter hypothesis.

## Introduction

The ability to walk on uneven terrain is important for uncompromised mobility and autonomy during daily life^1,2^. When the surface upon which a person walks is inconsistent and unpredictable, threats to balance during locomotion may be encountered. Such challenges can result in falls or a fear of falling that may deter individuals from participating in recreational and employment activities^3,4^. As many activities pursued from day to day will involve exposure to uneven surfaces to some extent, an inability or lack of confidence to tackle inconsistencies in terrain may be detrimental to overall quality of life^5^.

**Figure 1:**
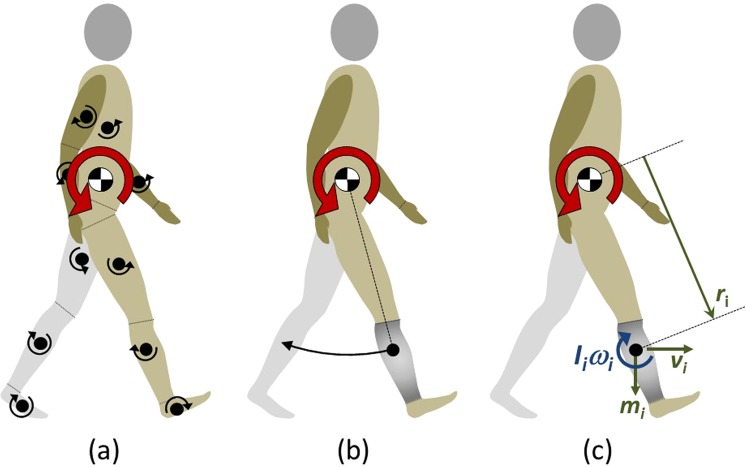
Whole-body angular momentum (red arrow) as the summation of the individual momenta of the segments (black arrows) about their centers of mass (**a**) and about the whole-body center of mass (**b**); computationally illustrated in the sagittal plane for the right lower leg segment in (**c**), where *I*_*i*_ and *ω*_*i*_ are the lower leg moment of inertia and angular velocity respectively, *r*_*i*_ is the distance between lower leg segment center of mass and whole-body center of mass, and *m* and *v*_*i*_ the lower leg mass and linear velocity, respectively, for the individual segment, *i*.

Maintaining balance during walking depends on the ability of the individual to effectively control the center of mass (COM) of the body with respect to the feet^6,7^. This occurs through the coordination of rotations of the segments of the body; via muscle action that is appropriately timed given the intrinsic demands of the task^8,9^. The whole-body angular momentum (*L*), which can be computed across the gait cycle, represents the sum of all rotational momenta of the segments acting about a set point, e.g. their combined COM (Fig. 1), and provides a quantification of the combined effect of their relative movements^8–10^.

The time rate of change of *L* is equal to the net external moment acting on the body. Thus, *L* at any instance is dependent on the action of external forces, which may be modulated by internally-produced muscular forces^8^, given the demands of a particular task. As such, the regulation of *L* may reveal the effectiveness of the rotational coordination produced by an individual given changes in the external environment and task demand, pertinent to mobility and fall risk. *L* must be actively generated in order to aid the performance of certain tasks, such as rising from a chair^11^ or ascending a slope^12^. In contrast, *L* is low in normal walking despite substantial values at segmental level^10,13^; less than 0.05 dimensionless units when normalised to mass, height and velocity. To put this into context, Herr & Popovic^10^ illustrated this to be as low as one sixth of the angular momentum of analogous point mass rotating rigid body systems^10^. These low values appear to be due to a balance of opposing segmental rotations; for example, about a vertical axis, the momenta of the legs are balanced by those of the upper body^10^. When external influences change during walking, changes must duly be made internally (i.e. via muscle activity) in order for the rotational momentum balance to be maintained^10,14^. A failure to constrain angular momentum in a particular direction could lead to excessive movement of the COM with respect to the base of support, resulting in destabilisation and a fall if adequate recovery measures are not employed to prevent one^15^.

For a known locomotor challenge, strategies may be actively employed to prevent loss of balance. For example, the average range of *L* during stair and slope descent has been shown to reduce on average in comparison to level walking^12,16^. Given that external forces would act to increase forward angular momentum on a decline, this finding implies the use of active control that over-compensates for this change in demand, but reduces the risk of a fall.

Such a strategy is arguably pre-emptive, however, and is possible due to the predictability of the demands of the task. Isolated perturbations during locomotion also act to increase *L* because they inherently involve a change in the external moments acting on the body^9,15^, leading to a requirement for the re-orchestration of movement if recovery is to be achieved^15^. These incidents are unpredictable by nature, precluding the employment of predictive strategies^6^ unless an individual is primed for such an event to occur. When responding to a perturbation, the covariance of the momenta of the segments has been shown to be more consistent than during normal walking, with action specific to the direction of perturbation^9^. This direction-specific strengthening in segmental coupling indicates that it is possible for appropriate, targeted adjustments to be rapidly made that can attenuate, although not eliminate, fluctuations in *L* induced by external challenges. It further supports the notion that the normal walking of unimpaired individuals is not as tightly constrained as it has the capacity to be; and, further, that fluctuations observed in walking patterns from stride to stride may be indicative of system adaptability^17,18^. It appears that the regulation of *L* in unimpaired individuals depends on what is required to efficiently meet the demands of the task, balanced with what is required to prevent destabilisation and a potential fall.

In the outdoor environment, the contours of the ground, and thus the point of action and direction of the ground reaction force, may be perpetually changing, such that every step is perturbed in a different way. As such, uneven terrain walking combines elements of the above tasks; it is *predictably unpredictable*. A higher *L* occurring as a result of induced imbalances in rotational momenta may result in destabilisation. Similarly, a higher *L variability* would lead to more instances that could result in destabilisation.

In order to *perfectly* regulate *L* when walking on such a surface, rapid, targeted alterations to movement dynamics would need to be made on a step-to-step basis, via changes to muscle action that are appropriate to each isolated encounter with the ground. To maintain movement fluidity, an instantaneous drop or increase in downhill slope, for example, might require *L* to be arrested to prevent a forward or lateral fall. An incline or increase in surface height might require *L* generation to raise the center of mass and efficiently maintain forward progression, or to achieve lateral weight transfer. Empirical data supports a capability to produce targeted responses to changes in the support surface; for example, during the stance phase of gait, experimentally-induced loading^19^ and unloading^20,21^ of the ankle extensors, simulating rapid dorsi- and plantarflexion, have been shown to result in marked soleus activity increases and decreases, respectively, in response. The extent to which *L* fluctuates, however, will depend on the coordination of rotations of all segments of the body in light of such changes. It is not currently known whether able-bodied individuals act to reduce fluctuations in *L* on uneven terrain, even though the means to provide rapid changes to movement may be available.

The purpose of this study was to explore the extent to which unimpaired individuals regulate *L* on uneven ground. It was hypothesised that on initial exposure to an uneven surface, both the range of *L* on average during stepping (*L*_R_) and its variability (standard deviation; v*L*_R_) would increase due to a lack of an appropriate strategy to attenuate the effect of changes in external forces on a step-to-step basis. It was further hypothesised that a reduction in both *L*_R_ and v*L*_R_ would be observed after a period of familiarisation with the surface, due to an improved ability to appropriately orchestrate the movements of the segments of the body given the external demands.

## Methods

### Participants

Eleven unimpaired male adults (mean ± sd; 24.4 ± 2.8 yrs, 78.3 ± 10.1 kg, 1.79 ± 0.09 m) were recruited to participate from the staff and study body of the University of Nebraska at Omaha. An a-priori power analysis performed in PASS (NCSS LLC, Kaysville, Utah, USA) based on a one-subject t-test using α = 0.0167 to account for multiple comparisons during post-hoc tests indicated that with a sample of 10 participants, effect sizes of 0.8 could be detected with 80% power at α = 0.05. Whilst there was no directly comparable data in the literature, this effect size was similar to that estimated from graphical results of a study exploring *L*_R_ on inclines and declines in unimpaired adults^12^. All procedures were approved by, and conducted according to the guidelines of, the University of Nebraska Medical Center Institutional Review Board, and all participants gave informed consent to participate. All participants had no reported history of ankle instability, joint laxity, knee ligamentous injury or knee instability, osteoarthritis or current musculoskeletal injury or pain. None had sustained a lower limb injury in the previous 12 months or had had surgery within the past 6 months. Participants were free from neurological disorders, movement disorders and cardiovascular disorders. All participants were able to walk comfortably for up to half an hour consecutively with no perceived fatigue and were familiar with treadmill walking and/or running.

### Procedures

Participants wore tight fitting clothing for motion capture purposes and were provided with flexible footwear comprised of lightweight low-profile canvas shoes, about which flexible shoe covers were securely affixed as part of a larger study (estimated footwear minimalist index: 60%; flexibility subscale: 5^22^). Reflective markers were placed at the legs, pelvis, torso, arms and head to enable full body kinematics to be captured, using configurations of anatomical markers for segment definition and tracking markers, based on Calibrated Anatomical Systems Technique^23^. Following a standing calibration trial, participants completed 5 minutes of treadmill walking at 1 m/s on a standard commercially available flat-belt treadmill (TRM 731, Precor, Woodinville, WA, USA), to gain familiarity with the footwear, equipment, imposed walking speed for the session and the environment.

**Figure 2:**
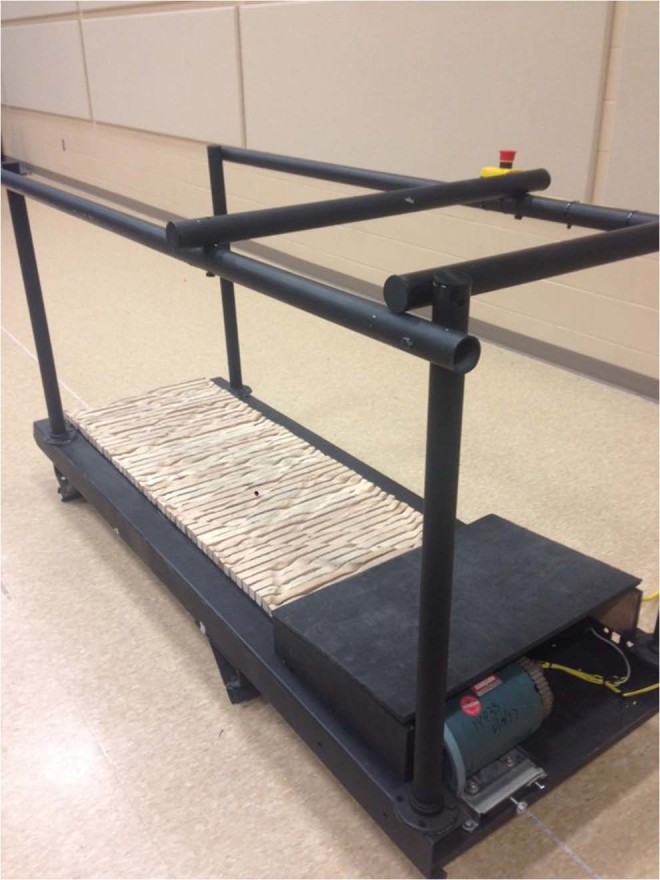
In-house built uneven terrain treadmill. Wooden slats, manually shaped to form a repeating pattern, were affixed to a standard treadmill belt. The pattern has four levels, with increments of approximately 7 mm, 7 mm and 8 mm.

Participants walked in two terrain conditions: flat (FT); on the treadmill described above, and uneven (UT); on an in-house modified uneven terrain treadmill of the same belt width (0.56 m) and average height (0.24 m). The latter device, illustrated in Fig. 2 has been previously shown to successfully invoke different midstance postures at each step, thus perpetually changing the direction of action of the ground reaction force during walking^24^. The surface, with a maximum depth of approximately 2.2 cm is sufficiently shallow to permit heel-toe gait, and the pattern is reflected about the center of the belt and offset to provide both feet with equal probability of encountering the same contours. A fixation cross was mounted on each treadmill to maintain visual consistency across trials.

Participants walked at a set speed of 1 m/s on the two treadmills. This speed was found to be comfortable on the UT in 17 healthy adults tested previously in our laboratory and was consistent with previous literature^25^. Predefining the walking speed was necessary because the protocol required assessment of the first steps on the uneven terrain. This precluded the use of standard means of determining a preferred speed prior to the start of the uneven terrain trial. Both trials lasted 12 minutes; permitting at least 5 minutes of walking to be performed^26^ prior to the post-exposure analysis. Trials commenced with the participant straddling the belt and stepping on when the belt had reached the target speed, to avoid any familiarisation with the terrain occurring during the acceleration period. Participants were asked to “walk naturally”, looking at the fixation cross. A rest period of at least 5 minutes was enforced between trials.

Kinematic data were acquired at 100 Hz^9^ using a 17-camera motion capture system (Motion Analysis Corp., Santa Rosa, CA, USA), and video data were captured for verification/quality assurance purposes. A 4th-order low pass Butterworth filter was applied to raw marker data. Cut off frequencies were determined using power spectral analysis as originally described by Winter^27^, and ranged from 7 Hz (head) to 12 Hz (feet). Foot contact events were estimated from the relative velocity of the pelvis and foot segments^28^.

### Computation

Angular momentum calculations were performed in Visual 3D (C-motion Inc, Germantown, MD, USA) from kinematic data, based on a 15-segment model comprised of pelvis, trunk, head, and bilateral feet, thighs, shanks, upper arms, lower arms and hands (see Supplementary Tables S1–S4 for full model details). For each segment, the COM location and moment of inertia were calculated based on Hanavan *et al*.^29^, with segment mass calculated as a percentage of body mass according to Dempster^30^, and whole-body COM was estimated from the combined position and masses of the individual segments. Individual segment momenta were computed from the sum of two components. The first quantifies the angular momentum of the segment about its own center of mass^31^. The second accounts for the influence of the mass and motion of the segment about a fixed point of reference^31^; in this case, the whole-body COM. About each axis, *L* was quantified through the summation of the two components of angular momenta of all of the segments, according to Eq. (1)^10,31^:

1 $$\overrightarrow{L}=\mathop{\sum }\limits_{i=1}^{n}[\overrightarrow{{I}_{i}}{\overrightarrow{\omega }}_{i}+\overrightarrow{{r}_{i}}\times {m}_{i}\overrightarrow{{v}_{i}}]$$

where, for each segment, *i*, $\overrightarrow{{I}_{i}}$ and ${\overrightarrow{\omega }}_{i}$ are segment moment of inertia and angular velocity vectors respectively, $\overrightarrow{{r}_{i}}$ is the vector distance between segment and whole-body center of masses, and *m* and $\overrightarrow{{v}_{i}}$ the segment mass and linear velocity vector respectively (illustrated in the sagittal plane in Fig. 1c). *L* for each participant was normalised to body mass and height to aid comparison across individuals and with previously reported values (note, data were inherently normalised to walking velocity due to the set speed of 1 m/s, rendering *L* a dimensionless quantity)^10^. *L* was calculated about the X, Y and Z axes of the global coordinate system corresponding to sagittal, coronal and transverse plane rotations respectively, with positive directions counterclockwise when viewing the body from the right side, front, and top.

*L* was calculated for each condition (FT, UT1 and UT2) and ensemble plots created for visualisation purposes. The range of *L* over the first 50% of each time-normalised gait cycle was computed (see e.g.^12,16,32^). This encompasses the initial double support period of gait, followed by the ipsilateral single support phase, and reveals the extent to which momentum is regulated throughout this period incorporating both positive and negative directions. *L*_R_ (mean range of *L*) and v*L*_R_ (standard deviation of *L* range) were calculated from 60 strides at the beginning and end of the UT trial (UT1 and UT2 respectively). The comparative data from FT were extracted from the final 60 strides of the 12-minute FT trial. Variables were computed for the limb the participant reported they would kick a ball with, assumed to be the dominant limb for our purposes.

### Statistical analysis

Statistical analysis on grouped data was performed in SPSS v24 (IBM Corp., Armonk, NY, USA). Normality of distribution was confirmed using Shapiro-Wilks tests and Q-Q plots. In order to test the hypotheses that *L* and *L* variability would be (1) increased initially on uneven ground and (2) decreased following familiarisation, variables *L*_R_ and v*L*_R_ were analysed using a 1-way repeated measures analysis of variance with main factor of condition at 3 levels (FT, UT1, UT2; α = 0.05). The comparison FT-UT1 was used to test the first hypothesis and UT1-UT2 and FT-UT2 to test the second. Eta-squared, η^2^, was calculated as a measure of effect size, which indicates the proportion of variance of each dependent variable (here *L*_R_, v*L*_R_) that may be attributed to the independent variable (treadmill condition). Post hoc Tukey HSD tests were performed when significant differences were identified.

Single subject analysis was performed as part of our post-hoc procedures to further elucidate any potential differences in individual responses, using the Model Statistic^33^. This test, performed for each participant individually, compares the difference in mean values computed from several trials under each test condition against a test statistic^33,34^. Comparisons of *L*_R_ across FT-UT1, UT1-UT2 and FT-UT2 were performed using all 60 strides (n = 60) and evaluated at α = 0.05. In order to determine whether variability of *L*_R_ of an individual subject differed across FT, UT1 and UT2, the standard deviation was computed within consecutive sets of 3 strides (strides 1 to 3, 4 to 6, etc.) yielding an n of 20 observations for each condition, and the same comparisons across conditions were performed.

## Results

### Average whole-body angular momentum range

**Figure 3:**
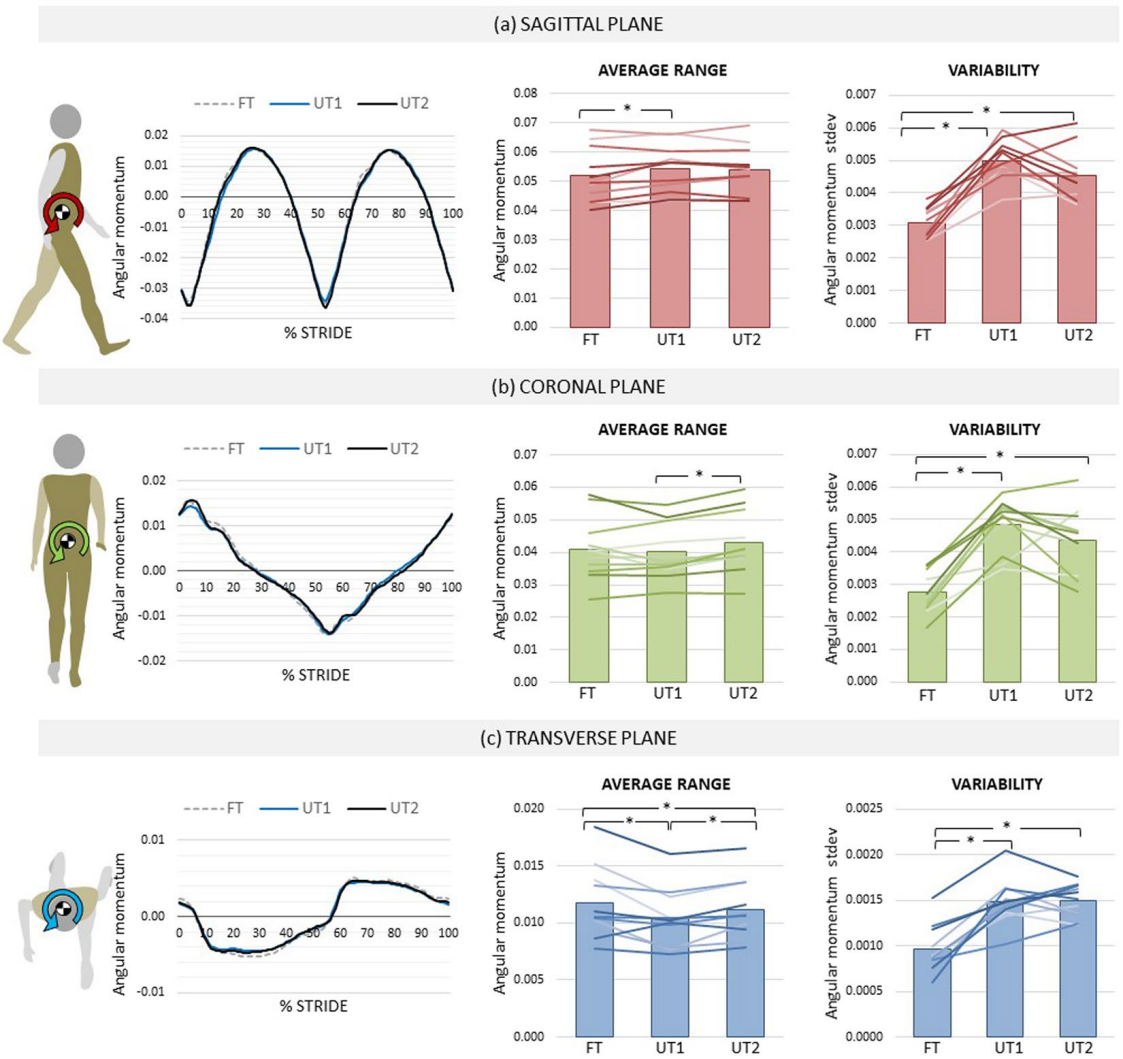
Whole-body angular momentum range and variability of range. Values computed over 60 strides on flat terrain (FT) and on uneven terrain at the beginning (UT1) and end (UT2) of a 12-minute trial in sagittal (**a**), coronal (**b**) and transverse (**c**) planes. Middle and right panes show grouped average results (bars) and individual subject results (line). Left panes show stride-normalised grouped results (n = 11). Range calculated across non-shaded regions. *Significant at α = 0.05.

Sagittal plane *L*_R_ showed a significant main effect of condition (F_2,20_ = 4.529, p = 0.024, η^2^ = 0.312) with post hoc tests revealing a significant increase (p < 0.05) between FT and UT1. Single subject analysis revealed that six individuals had a significantly greater *L*_R_ on first exposure to the UT in comparison to FT, with all but one other showing non-significant differences. Ensemble profiles suggest that the difference occurred during the early stance negative peak (Fig. 3a; left panel). Approximately half (n = 5) of the participants showed a change between UT1 and UT2, however the changes observed were largely balanced (Fig. 3a; middle panel). At UT2 six of the 11 participants had a significantly higher *L*_R_ than that observed on FT.

A significant difference in *L*_R_ was observed in the coronal plane (F_2,20_ = 5.267, p = 0.015, η^2^ = 0.345). Pairwise comparisons showed an increase from UT1 to UT2 (p < 0.05). From FT to UT1 three participants increased and three decreased *L*_R_, potentially explaining the lack of change at grouped level. In contrast, the majority of participants (n = 9) showed an increase in *L*_R_ from UT1 to UT2, and 6 retained a higher *L*_R_ at UT2 in comparison to FT (Fig. 3b; middle panel).

There was a significant change in average transverse plane *L*_R_ (F_2,20_ = 6.647, p = 0.006, η^2^ = 0.399). Post hoc tests revealed a significant decrease from FT to UT1 (p < 0.01), an increase from UT1 to UT2 (p < 0.01) and a decrease from FT to UT2 (p < 0.05). Single subject analysis largely supports these results with 8 participants decreasing *L*_R_ at UT1 and 5 increasing (vs 0 decreasing) *L* from UT1 to UT2, however 4 participants retained a significant decrease in *L*_R_ at UT2 in comparison to FT and 1 an increase. Of note, ensemble profiles reveal an attenuation of the negative *L*_R_ from approximately 15% of the stride (Fig. 3c; left panel). The observed power was 70%, 77% and 86% for sagittal, coronal and transverse plane *L*_R_, respectively.

### Whole-body angular momentum variability

There were significant differences in v*L*_R_ in all planes across conditions (Sagittal − F_2,20_ = 44.886, p < 0.001, η^2^ = 0.818; Coronal − F_2,20_ = 42.227, p < 0.001, η^2^ = 0.809; Transverse − F_2,20_ = 40.125, p < 0.001, η^2^ = 0.800). Post hoc tests revealed that in all planes significant differences existed between FT-UT1 and FT-UT2 (Fig. 3a–c; right panels). In all cases, variability was increased on the UT, and there was no change from UT1-UT2. Individual results support these findings with the majority of participants following these trends in the sagittal and coronal planes. Only two participants showed evidence of a decrease in v*L*_R_; and this was in the coronal plane. An observed power greater than 99% was attained in all three planes.

## Discussion

On immediate exposure to uneven terrain, the changes in *L*_R_ observed in comparison to FT were not consistent in all three planes (sagittal, coronal, transverse). Our first hypothesis, i.e. that on initial exposure to an uneven surface, *L*_R_ would increase due to a lack of an appropriate strategy to attenuate the effect of changes in external momentum on a step-to-step basis, was therefore only partially supported. The apparent difference across planes is consistent with other studies that explore the effect of perturbing gait or changing task demands on *L* (e.g.^9,35^).

The increase in sagittal plane *L*_R_ on UT in comparison to FT, as demonstrated in both the grouped results and the majority of individual subject results, supports our hypothesis. The increase in momentum primarily responsible for the increase in range (Fig. 3a) appeared to be isolated to the loading response phase of the gait cycle. This may imply that the dynamics have already been adjusted appropriately by the end of this early contact phase. In the coronal plane, grouped results showed no significant difference on initial exposure (FT-UT1), although significant results were observed at the individual level in many cases, indicating differing responses across participants.

The most uniform finding for our first comparison (FT-UT1) was a reduction in *L*_R_ in the transverse plane. This was supported by both grouped and individual results, and refuted our first hypothesis. Evident from Fig. 3(c) (left panel) the reduction in range occurs in the period from 15–50% of the gait cycle, which is within the single support phase of the contralateral limb, extending to the subsequent foot contact event. As *L*_R_ would be expected to increase in a perturbing environment^9^, a reduction in *L*_R_ might suggest that motion about the vertical axis was actively controlled during single limb stance, either by a reduction in rotational momenta of one or more segments or a change in the coupling between them^9^. This is consistent with previous studies that have examined transverse plane *L* during challenges to gait^9,36–38^. Such a strategy could reduce the likelihood of a crossover step, or of an abrupt misalignment of the body with the plane of progression that might prompt a fall over the lateral border of the foot. Alternatively, this observation may be related to a concomitant reduction in step length^24^, proposed to be a conservative mechanism to aid dynamic balance, which affects momenta due to a change in the effect of the ground reaction force^12^. Should this be the case, it remains unclear whether *L*_R_ is reduced as a consequence of a change in step length or actively controlled to reduce it.

The increase in variability observed at UT1 similarly supports our first hypothesis and would indicate that whatever strategy for walking on uneven ground is immediately employed, it cannot fully account for the changing contours beneath the feet. That nearly half of the group did not show a significant difference in transverse plane v*L*_R_, however, might again indicate an active restriction of movement in that plane, consistent with the decrease in average values. Again, the source of this restriction warrants further exploration.

We anticipated that *L*_R_ and v*L*_R_ would reduce with familiarisation to the surface. This second hypothesis was refuted in all planes. No change in *L*_R_ was observed in the sagittal plane over time, despite the increase from FT-UT1, and on an individual level the differences between UT1-UT2 (and FT-UT2) were sparse and in both directions, indicating the lack of a common response. In contrast, both coronal and transverse plane *L*_R_ increased on average from UT1-UT2. This increase in *L*_R_ at group level was supported by the individual results of majority of participants in the coronal plane (n = 9) and approximately half in the transverse plane (n = 5). These increases may be suggestive of a relaxation of the restrictions initially placed on the available degrees of freedom given familiarity with the task^39,40^. The apparent relaxation of internally imposed constraints in the coronal and transverse planes at UT2 may, further, indicate that the immediate solution adopted is not an optimal one. This is consistent with an increase in energy expenditure on uneven terrain that has been previously reported^25^, and also may be associated with a lack of system flexibility. Grouped *L*_R_ values in the transverse plane at UT2 on average remained lower than those observed at FT. That several participants (n = 4) retained a lower value of transverse plane *L*_R_ at UT2 might allude to differences in motor learning ability, or to persistent apprehension in some individuals that prevents them from releasing degrees of freedom.

Contrary to our second hypothesis, the finding that there was no change in v*L*_R_ in any of the grouped comparisons over time, nor in most of the individual cases regardless of plane, similarly indicates that participants did not learn nor act to precisely adapt their movement on a step-to-step basis in response to each interaction with the terrain. Whether this is because they were unable to or that there was no need to is unclear. It is plausible that for able-bodied individuals this level of variability is tolerable, given an awareness of their own constraints and ability to correct more extreme deviations when required. Further, the risk of an incident of destabilisation occurring with higher *L*_R_ and v*L*_R_ may have been insufficient to warrant the adoption of greater, potentially energetically costly, compensatory strategies. The contours of the treadmill belt in the present study were designed to permit foot-flat at every step, and it is possible that a more undulating or unpredictable surface could have produced different results.

The results of this study suggest that unimpaired adults are able to appropriately orchestrate their intersegmental movement on uneven ground, although do not completely ameliorate its effect. Further, although on a step-to-step basis their dynamics remain more variable over time, their movement becomes less tightly constrained as they become more familiar with the terrain. *L* is dependent on the magnitudes of individual segment momenta and the coordination between the movements of the segments, therefore there may be many strategies for regulating it. The observation of differences at individual level despite all participants being fully able-bodied, supports the assertion that individuals may adopt different strategies for walking on uneven terrain. The source of the differences may be explored by examining contributions to *L* or *L*_R_ at segmental level, and the coordination across limbs and between body segments.

Both sensory and mechanical deficits may have a profound effect on the ability of the individual to produce appropriate movement solutions in a given environmental context. The strategies employed on UT therefore warrant further investigation. If, for example, a reduction in transverse plane *L* is a necessary initial strategy to permit familiarisation and does not merely occur secondary to changes in foot placement, an inability to control rotations via targeted muscle action would preclude an individual from being able to achieve this. In individuals with limited lower limb range of motion or amputation, an inability to adjust compliance of the foot or produce an appropriately timed push off, could prevent this initial control from being achieved, leading to greater destabilisation on first encounter.

The effect of limb dominance was not explored in the present study. The incorporation of this analysis in a future larger study may be revealing of any specific roles of the dominant and non-dominant lower limb in arresting and generating whole-body angular momentum. Knowledge of such differences may be valuable for providing a prognosis and evaluating fall risk in individuals who experience unilateral injury or amputation and are unable to exploit either the dominant or non-dominant limb.

Whilst the uneven terrain treadmill utilised in this study permitted the analysis of extended bouts of walking, it may have induced differences in movement due to spatial restrictions related to belt length and width, and the fixed treadmill speed. That our results from the FT treadmill, which has similar dimensions to the UT treadmill, were similar to previously published values obtained during overground walking provides further confidence in our paradigm. However, it cannot be excluded that movement was influenced by the terrain and that results obtained on a larger, fixed-speed treadmill or extended overground walking surface may refute the findings of this study. A further limitation lies in the observation of only healthy, young male participants. Whilst this will have increased sample homogeneity it may limit the generalisability of our results, and comparative analyses with individuals with substantially different body shapes, e.g. females, individuals with obesity, may in themselves may be revealing.

## Conclusion

In unimpaired male adults, *L*_R_ in the sagittal plane and v*L*_R_ in all planes increased on uneven terrain implying a lesser degree of *L* regulation, given perturbations induced by the environment. In contrast, there was a reduction in transverse plane *L*_R_, consistent with immediate employment of a different strategy for this plane, related to the restriction of available degrees of freedom. Over time, coronal and transverse plane *L*_R_ increased, indicating a learning effect that may be associated with the release of degrees of freedom. The initial increases in v*L*_R_ in all planes and in sagittal plane *L*_R_ were not reduced, however, implying that participants either were unable to, or do not need to, completely eliminate the influence of the terrain on a step-to-step basis. The investigation of angular momentum during uneven terrain walking in individuals with mechanical or sensory deficit could allow us to assess fall risk and provide further insight into activity avoidance.

## Supplementary information

Supplementary information

## Data Availability

The datasets generated analysed during the current study are available from the corresponding author on reasonable request.
