# Supplementary material for: Changes in human walking dynamics induced by uneven terrain are reduced with ongoing exposure, but a higher variability persists
Source: Sci Rep. 2019 Nov 27;9:17664. doi: 10.1038/s41598-019-54050-z (PMC6881352; doi:10.1038/s41598-019-54050-z)
Supplement: Supplementary file 1 — Supplementary information [file 41598_2019_54050_MOESM1_ESM.docx]

Changes in human walking dynamics induced by uneven terrain are reduced with ongoing exposure, but a higher variability persists

Jenny A Kent, Joel H Sommerfeld, Nicholas Stergiou

##### **Supplementary Information**

##### FULL BODY MODEL – DESCRIPTION

**Supplementary Table S1. Model Fundamentals**

| **Design** | In-house developed full body model and location protocol. |
| --- | --- |
| **Degrees of freedom** | All joints/segments : 6 |
| **Optimisation** | Segment optimisation - Software generic.  See Visual 3D documentation at  *http://www.c-motion.com/v3dwiki/index.php?title=Six_Degrees_of_Freedom* and ^1^ |
| **Kinetic calculations** | Mass, moments of inertia and center of gravity calculated according to ^2^ (see Visual 3D documentation at *http://c-motion.com/v3dwiki/index.php/Segment_Geometry* |

**Supplementary Table S2. Landmarks**

| **Landmark ID**  **(^bilateral/side)** | **Location** | **Location method** | **Function** | | **Type** |
| --- | --- | --- | --- | --- | --- |
|  |  |  | Definition | Tracking |  |
| ^ASI | Superficial on skin surface such that marker body is anterior to prominent anterior edge of iliac crest. | Palpation | Yes | Yes | Marker |
| ^PSI | Superficial to palpable prominence at posterior edge of iliac crest. | Palpation | No | Yes | Marker |
| ^ASI2 | Superficial and lateral to proximal border of iliac crest. | Palpation | No | Yes | Marker |
| ^VPSI | PSI projected by marker radius perpendicular to contralateral PSI in plane connecting PSIs and mid point between ASIs. | Calculation | Yes | No | Derived |
| ^VASI | ASI projected by marker radius perpendicular to contralateral ASI in plane connecting ASIs and mid point between PSIs. | Calculation | Yes | No | Derived |
| ^HJC | Hip joint centre – at the following distances from mid-point between VASI markers (Pelvis origin) in pelvis coordinate system (see Pelvis):  AP (in mm) = -0.24PD - 9.9  ML (in mm) = 0.28PD + 0.16PW +7.9  Axial (in mm) = -0.16PW-0.04LL-7.1  Where PD = Pelvic depth: the distance between the mid points of VASIs and VPSIs  PW = Pelvic width : distance between left and right VASIs  LL = Leg Length : distance between the ASI and VANM via the VKNM ^3^ | Calculation | Yes | No | Derived |
| ^TRO | Lateral and superficial to the centre of the palpated prominence of the greater trochanter when standing in calibration posture. | Palpation | No | Yes | Marker |
| ^TH1 | Anteriorly on thigh, approximately 1/3 distance between hip and knee. | Visualisation | No | Yes | Marker |
| ^TH2 | Anteriorly on thigh, 50mm (approx.) above the patella when relaxed in standing. | Visualisation | No | Yes | Marker |
| ^TH3 | Laterally on thigh, approximately mid distance between hip and knee | Visualisation | No | Yes | Marker |
| ^TH4 | Lateral on thigh, between TH3 and TRO | Visualisation | No | Yes | Marker |
| ^KNL | At the bony prominence of the lateral femoral condyle, to form the lateral end of a ‘knee axis’ with ^KNM. | Palpation | Yes | Yes | Marker |
| ^KNM | At the bony prominence of the medial femoral condyle, to form the medial end of a ‘knee axis’ with ^KNL. | Palpation | Yes | No | Marker* |
| ^VKNL | KNL projected by marker radius in direction of KNM. | Calculation | Yes | No | Derived |
| ^VKNM | KNM projected by marker radius in direction of KNL. | Calculation | Yes | No | Derived |
| ^KJC | Mid point between KNL and KNM. | Calculation | Yes | No | Derived |
| ^SHA1-4 | Set of four markers placed anteriorly and posteriorly on lower 1/3 of the shank positioned to avoid excessive rotation due to individual tendon/muscle protrusion on dorsi/plantar flexion. | Visualisation | No | Yes | Marker |
| ^ANL | At most lateral point on lateral malleolus, to form the lateral end of the ‘ankle’ axis with ^ANM. | Palpation | Yes | No | Marker |
| ^ANM | At most medial point on medial malleolus, to form the medial end of the ‘ankle’ axis with ^ANL. | Palpation | Yes | No | Marker^*^ |
| ^VANL | ANL projected by marker radius in direction of ANM. | Calculation | Yes | No | Derived |
| ^VANM | ANM projected by marker radius in direction of ANL. | Calculation | Yes | No | Derived |
| ^AJC | Mid point between ANL and ANM |  |  |  |  |
| ^TOE | At a point approximating position of second metatarsal head on dorsum of shoe | Palpation /Visualisation | Yes | Yes | Marker |
| ^MT1 | On dorsum of shoe at a point approximating position of first metatarsal head. | Palpation /Visualisation | Kinetic only | Yes | Marker |
| ^MT5 | On dorsum of shoe at a point approximating position of fifth metatarsal head. | Palpation /Visualisation | Kinetic only | Yes | Marker |
| ^FT3 | On dorsum of shoe proximal to MT1 and MT5. | Palpation /Visualisation | No | Yes | Marker |
| ^HEE | On heel counter, approximately 15mm from ground, centrally when viewing from a posterior position along the long axis of the shoe to form the longitudinal axis of the foot with TOE | Measurement /Visualisation | Yes | Yes | Marker |
| ^TOEvert | Projection of TOE onto laboratory floor. | Calculation | Yes | No | Derived |
| ^HEEvert | Projection of HEE onto laboratory floor. | Calculation | Yes | No | Derived |
| ^HEElat | Projection of HEEvert laterally in pelvis coordinate system. | Calculation | Yes | No | Derived |
| STRN | Anterior and superficial to sternal notch | Palpation | Yes | Yes | Marker |
| XYPH | Superficial to xyphoid process | Palpation | Yes | Yes | Marker |
| C7 | Superficial to seventh cervical vertebra | Palpation | Yes | Yes | Marker |
| LUM | Lumbar region, superficial to the spine at point of maximum curvature. | Palpation | Yes | Yes | Marker |
| LowerTorso | Mid point between XYPH and LUM | Palpation | Yes | No | Marker |
| UpperTorso | Mid point between STRN and C7 | Palpation | Yes | No | Marker |
| ^SHA | Anterior to the approximate shoulder joint centre. | Palpation/ Visualisation | Yes | Yes | Marker |
| ^ACR | Vertically above the acromium process | Palpation | Yes | No | Marker* |
| ^SJC | Projection of SHA onto the plane made by R & LACR and the vertical projection of RACR. | Calculation | Yes | No | Derived |
| ^UPA | On the posterior upper arm approximately half way between the shoulder and elbow. | Visualisation | No | Yes | Marker |
| ^ELL | Superficial to the lateral condyle, placed with the arm hanging to the side of the body | Palpation | Yes | Yes | Marker |
| ^ELM | Approximately superficial to the medial condyle, placed with the arm hanging to the side of the body to form an axis through the elbow with ELL | Palpation | Yes | No | Marker* |
| ^VELL | ELL projected by marker radius in direction of ELM | Calculation | Yes | No | Derived |
| ^VELM | ELM projected by marker radius in direction of ELL | Calculation | Yes | No | Derived |
| ^EJC | Mid point between ELL and ELM | Calculation | Yes | No | Derived |
| ^HA1 | Between 1^st^ and 2^nd^ metacarpals, approximately 15mm from the metacarpal heads. | Palpation | Yes | Yes | Marker |
| ^HA2 | On the radial process of the wrist, to form a ‘wrist axis’ through the joint with HA3. | Palpation | Yes | Yes | Marker |
| ^HA3 | On the ulnar process of the wrist, to form a ‘wrist axis’ through the joint with HA2. | Palpation | Yes | Yes | Marker |
| ^VHA2 | HA2 projected by marker radius in direction of HA3 | Calculation | Yes | No | Derived |
| ^VHA3 | HA3 projected by marker radius in direction of HA2 | Calculation | Yes | No | Derived |
| ^FIN | HA1 projected distally 0.5*distance between HA1 and midpoint of HA2 and HA3, along the line formed by HA1 and then midpoint of HA2 and HA3 | Calculation | Yes | No | Derived |
| ^FHD | On band approximately 2 cm above brow line, vertically above corner of eye | Visualisation | Yes | Yes | Marker |
| ^BHD | On band approximately 2 cm laterally of bony protrusion on back of head | Palpation/ Visualisation | Yes | Yes | Marker |
| ^HEC | Mid point of line between FHD and BHD | Calculation | Yes | No | Derived |
| LFHD_proj | LFHD projected backwards in global coordinate system by 0.05m | Calculation | Yes | No | Derived |
| ^HEP | HEC projected (upwards) onto plane defined by RFHD, LFHD and LFHD_proj | Calculation | Yes | No | Derived |

* Marker removed following static calibration trial

**Supplementary Table S3. Segment definitions**

| **Segment (^bilateral/**  **side)** | **Landmarks** (derived landmarks in parentheses) | **Origin** | **Axes** | | | **Geometry***  **Joint radii** | **Tracking markers** |
| --- | --- | --- | --- | --- | --- | --- | --- |
|  |  |  | **Flex/ext** | **Add/abd** | **Axial** |  |  |
| Pelvis (V3D Composite^a^) | LASI, RASI, LPSI, RPSI, LAS2, RAS2, (LVASI), (RVASI), (LVPSI), (RVPSI) | Midpoint between LVASI and RVASI markers | Parallel to line from origin to RVASI | Orthogonal to the flex/ext and axial axes. | Perpendicular to the plane defined by LVASI, RVASI & the midpoint between LVPSI and RVPSI | See ^a^ | LASI, RASI, LPSI, RPSI, LAS2, RAS2, VSAC |
| ^ Thigh | (VKNL), (VKNM), TRO, TH1, TH2, TH3, KNL (HJC) | HJC | Perpendicular to axial axis in plane defined by HJC, VKNL and VKNM | Orthogonal to axial and flex/ext axes | Line joining HJC and midpoint between VKNL and VKNM | Proximal: half distance between RTRO and LTRO | TRO, TH1-4, KNL^b^ |
| ^ Shank | (KJC), (VANL), (VANM), ANL, SK1-4 | KJC | Perpendicular to axial axis in plane defined by KJC, VANL and VANM | Orthogonal to Axial and Flex/Ext axes | Line joining KJC and midpoint between VANL and VANM | Proximal: half distance between VANL and VANM | SK1-4, KNL, ANL^b^ |
| ^ Foot | (AJC), (VANL), TOE, LHL, HEE, MT5 | AJC | Perpendicular to axial axis in plane defined by AJC, VANL and TOE | Orthogonal to Axial and Flex/Ext axes | Line joining AJC and TOE | Proximal : distance between AJC and VANL, Distal : half distance between MT1 and MT5 | TOE, HEE, MT1, MT5, FT3 |
| ^ KMAT Foot | (HEEvert), (TOEvert), (HEElat), TOE, LHL, HEE, MT5 | HEEvert | Perpendicular to axial axis in plane defined by HEEvert, TOEvert and HEElat | Line joining HEEvert and TOEvert | Orthogonal to Add/Abd and Flex/Ext axes | NA | TOE, HEE, MT1, MT5, FT3 |
| Thorax | (UpperTorso), (LowerTorso), STRN, XYPH, C7, LUM. | Upper Torso | Perpendicular to axial axis, orthogonal to plane defined by UpperTorso, LowerTorso and STRN. | Orthogonal to Axial and Flex/Ext axes | Line joining UpperTorso and LowerTorso | Proximal : half distance between RSJC and LSJC  Distal : half distance between RASI and LASI | STRN, XYPH, C7, LUM |
| ^ Upper Arm | (SJC), UPA, (VELL), (VELM), SHA, ELL | SJC | Perpendicular to axial axis in plane defined by SJC, VELL and VELM | Orthogonal to Axial and Flex/Ext axes | Line joining SJC to mid point between VELL and VELM | Proximal : distance between SJC and ACR | SHA, UPA, ELL |
| ^ Forearm | (EJC), (VHA2), (VHA3), ELL, HA2, HA3 | EJC | Perpendicular to axial axis in plane defined by EJC, VHA2 and VHA3 | Orthogonal to Axial and Flex/Ext axes | Line joining EJC to mid point between VHA2 and VHA3 | Proximal : distance between VELL and VELM | ELL, HA2, HA3 |
| Hand | (FIN), HA1, HA2, HA3, (VHA2), (VHA3) | Midpoint between VHA2 and VHA3 | Perpendicular to axial axis in plane defined by FIN, VHA2 and VHA3 | Orthogonal to Axial and Flex/Ext axes | Line joining FIN and midpoint between VHA2 and VHA3 | Distal : half distance between VHA2 and VHA3 | HA1, HA2, HA3 |
| Head | (RHE),(LHE), (Upper Torso), LFHD, RFHD, LBHD, RBHD | Mid point between RHE and LHE | Perpendicular to axial axis in plane defined by RHE, LHE and Upper Torso | Orthogonal to Axial and Flex/Ext axes | Line joining mid point between RHE and LHE and Upper Torso | Proximal : Half distance between LFHD and LBHD minus 1 marker diameter. Depth : half distance between LFHD and LBHD minus 1 marker diameter | LFHD, RFHD, LBHD, RBHD |

^a^ See <http://www.c-motion.com/v3dwiki/index.php?title=V3D_Composite_Pelvis>

^b^ Data from repeatedly obscured markers excluded. Segments tracked with at least 3 non-collinear markers including at least 1 anterior and 1 posterior placed marker.

**Supplementary Table S4. Joint definitions / rotation sequences**

| **Joint / Segment angle name^a^ (*bilateral)** | **Segment^b^** | **Reference segment^b^** | **Cardan sequence** | **Positive direction** |
| --- | --- | --- | --- | --- |
| Pelvis | Pelvis | Laboratory | Flex/ext - add/abd - int/ext rotation | Tilt – anterior  Obliquity – right down  Twist – right forwards |
| Hip* | Thigh | Pelvis | Flex/ext - add/abd - int/ext rotation | Flexion  Adduction  Internal rotation |
| Knee* | Shank | Thigh | Flex/ext - add/abd - int/ext rotation | Flexion  Adduction  Internal rotation |
| Ankle* | Foot | Shank | Flex/ext-add/abd-inv/ev rotation | Dorsiflexion  Adduction  Inversion |
| Virtual Ankle* | Virtual Foot | Virtual Shank | Flex/ext-inv/ev-add/abd | Dorsiflexion  Inversion  Adduction |

^a^ Virtual segments are used for joint kinematics only.

^b^ Angles are calculated within the co-ordinate system of the reference segment.

**Supplementary Table S5. System**

| **Capture system / software** | Motion Analysis Corporation ^a^: 17-camera Raptor/ Cortex version 6 ^a^ |
| --- | --- |
| **Medium** | Passive retro-reflective markers : 12.7mm with thin fabric base |
| **Sampling frequency** | 100Hz |

**Supplementary Table S6. Processing**

|  | **Software** | **Details** |
| --- | --- | --- |
| **Eventing** | Visual 3D^b^ | Kinematic algorithm (velocity-based, from ^4^) |
| **Filtering** | Visual 3D^b^ | 7Hz-12Hz 4^th^ order Butterworth, by marker ^5^ |
| **Interpolation** | Cortex ^a^ | Cubic spline / software-based virtual join ^a^ |

^a^ Motion Analysis Corporation, Santa Rosa, CA, USA

^b^ C-Motion, Germantown, MD, USA

^c^ Mathworks, Natick, MA, USA

**References**

^1^ Spoor CW. Rigid body motion calculated from spatial co-ordinates of markers. J. Biomech 1980; 13: 391-393.

^2^ Hanavan Jr, EP. A mathematical model of the human body (No. AFIT-GA-PHYS-64-3). Air Force Aerospace Medical Research Lab Wright-Patterson Afb Oh, 1964.

^3^ Harrington ME, Zavatsky AB, Lawson SE, Yuan Z, Theologis TN. Prediction of the hip joint centre in adults, children, and patients with cerebral palsy based on magnetic resonance imaging. J Biomech. 2007;40(3):595-602.

^4^ Zeni Jr, JA., Richards, JG, & Higginson, JS. Two simple methods for determining gait events during treadmill and overground walking using kinematic data. Gait Posture 2008, 27(4), 710-714.

^5^ Giakas, G. Power spectrum analysis and filtering. Innovative Analyses of Human Movement, Champaign, IL: Human Kinetics, 2004.
